# Case report: A novel homozygous variant in ZP3 is associated with human empty follicle syndrome

**DOI:** 10.3389/fgene.2023.1256549

**Published:** 2023-10-16

**Authors:** Na Kong, Qian Xu, Xiaoyue Shen, Xiangyu Zhu, Guangyi Cao

**Affiliations:** ^1^ Center for Reproductive Medicine and Obstetrics and Gynecology, Nanjing Drum Tower Hospital, Affiliated Hospital of Medical School, Nanjing University, Nanjing, China; ^2^ Center for Molecular Reproductive Medicine, Nanjing University, Nanjing, China; ^3^ Guangdong Provincial Key Laboratory of Reproductive Medicine, Guangzhou, China

**Keywords:** ZP3, empty follicle syndrome, oocyte, reproduction, infertility

## Abstract

Empty follicle syndrome (EFS) is a rare condition in female infertility. It is characterized by the inability to retrieve oocytes from visibly large, normally developing follicles in the ovaries, despite ovarian stimulation. The genetic factors contributing to this syndrome remain unclear. This study focused on patients who underwent three consecutive ovarian stimulation procedures for oocyte retrieval but experienced unsuccessful outcomes, despite the presence of observable large follicles. Ultrasound examinations were conducted to assess follicular development during each procedure. In order to investigate potential genetic causes, we performed whole exome sequencing on peripheral blood samples from the patient. Interestingly, we identified that this patient carries a homozygous mutation in the *ZP3* genes. Within the *ZP3* gene, we identified a homozygous variant [NM_001110354.2, c.176T>A (p.L59H)] specifically located in the zona pellucida (ZP) domain. Further analysis, including bioinformatics methods and protein structure modeling, was carried out to investigate the conservation of the ZP3^L59H^ variant across different species. This homozygous variant exhibited a high degree of conservation across various species. Importantly, the homozygous ZP3^L59H^ variant was associated with the occurrence of empty follicle syndrome in affected female patients. The homozygous ZP3^L59H^ variant represents a newly discovered genetic locus implicated in the development of human empty follicle syndrome. Our findings contribute to a deeper understanding of the role of zona pellucida-related genes in infertility and provide valuable insights for the genetic diagnosis of female infertility.

## Introduction

Empty follicle syndrome (EFS) refers to a condition where no oocytes can be retrieved from apparently large, normally growing, fluid-filled follicles in the ovaries after stimulation. EFS can be classified into two main types based on circulating HCG levels: the “genuine” form (gEFS) and the “false” form (f-EFS) (Kim and Jee, 2012; Revelli et al., 2017). Although this condition has been reported in the literature since 1986 (Coulam et al., 1986), the underlying mechanisms remain unclear. As a severe defect in oocyte development, some studies suggest that the absence of genes encoding zona pellucida (ZP)-related proteins may be implicated in this phenomenon (Chen et al., 2017; Yuan et al., 2017; Sun et al., 2019; Xu et al., 2020; Zhang et al., 2021; Hou et al., 2022; Jia et al., 2022; Shen et al., 2022; Zhang et al., 2022; Sun et al., 2023).

The zona pellucida (ZP) is an extracellular glycoprotein matrix that accompanies the growth of oocytes and remains intact around the embryo until hatching (Matzuk et al., 2002; Avella et al., 2014). Within the primary follicle, the ZP physically separates the oocyte from the surrounding granulosa cells, aiding in oocyte growth and meiotic arrest (Kalab et al., 1993; Hasegawa and Koyama, 2007). Throughout the process of follicle development, various cellular factors present in the follicular fluid continuously modify the zona pellucida (ZP) as part of its preparation for fertilization. Some examples of these factors include lactoferrin, OVGP1, and glycolytic enzymes (Kalab et al., 1991; Carino et al., 2001; Serres et al., 2008; Moros-Nicolas et al., 2021). During the process of fertilization, the sperm and oocyte encounter each other. The zona reaction serves to prevent polyspermy and facilitates species-specific binding of the sperm (Wassarman, 2008; Litscher et al., 2009). Finally, the ZP protects the embryos as they traverse the female reproductive tract toward implantation in the uterus.

The human ZP is primarily composed of four proteins (hZP1, hZP2, hZP3, hZP4). These ZP-related proteins exhibit structural conservation and possess a signal sequence, ZP domain (ZPD), consensus furin cleavage site (CFCS), and transmembrane domain (TMD). The trefoil domain is found only in ZP1 and ZP4 (Jovine et al., 2002; Litscher and Wassarman, 2020; Wassarman and Litscher, 2021). Interestingly, hZP3 contains a region of 45 amino acids between the ZPD and the CFCS, which includes the sperm-binding site (Wassarman, 1987; Williams and Wassarman, 2001). In contrast, hZP1, hZP2, and hZP4 have only 3 to 5 amino acids in that region. Notably, this region of the hZP3 polypeptide also harbors four conserved cysteine residues in close proximity, suggesting the potential formation of two intramolecular disulfide bonds (Litscher and Wassarman, 2020). On the other hand, the mouse ZP comprises three proteins, mZP1, mZP2, and mZP3. ZP genes are exclusively expressed in female mice. Male mice homozygous for ZP null mutations remain fertile. However, females homozygous for ZP2 or ZP3 null mutations produce oocytes lacking the ZP, leading to complete infertility. This infertility arises from the absence of developing oocytes in the ovaries and the lack of ovulated eggs in the oviduct (Liu et al., 1996; Rankin et al., 1996; Rankin et al., 2001). Although female mice with homozygous mutations in ZP1 are fertile, they exhibit a decreased number of offspring due to reduced numbers of preimplantation embryos in the uterus (Rankin et al., 1999). In the context of assisted reproductive treatments, the morphological aspects of the ZP (including color, thickness, refractive index, etc.,) are valuable indicators of oocyte quality. Understanding the genetic mechanisms that influence ZP morphology plays a crucial role in improving the success rates of assisted reproduction. Recent studies have reported associations between abnormal or absent ZP morphology and mutations in hZP1, hZP2, and hZP3 (Huang et al., 2014; Chen et al., 2017; Yuan et al., 2017; Sun et al., 2019; Zhou et al., 2019; Xu et al., 2020; Zhang et al., 2021; Hou et al., 2022; Jia et al., 2022; Zhang et al., 2022).

In this study, we identified a novel homozygous variant [NM_001110354.2, c.176T>A (p. L59H)] located at the N-terminus of the ZP domain in the *ZP3* gene through whole-exome sequencing. Despite the presence of large follicles, this patient failed to retrieve any oocytes after three rounds of ovarian stimulation. Subsequent bioinformatic analysis provided further confirmation of the impact of this new mutation on the ZP3 protein.

## Materials and methods

### Ethics approval

Approval for this study was obtained from the Ethics Committee of Nanjing Drum Tower Hospital (2021-384-01). All embryos analyzed in this study were obtained from the Center for Reproductive Medicine, Nanjing Drum Tower Hospital, Affiliated Hospital of Medical School, Nanjing University. Informed consent was obtained from all participants consenting to the collection of clinical samples relevant to this study.

### Whole-exome sequencing (WES) and variant analysis

Genomic DNA was extracted from peripheral blood samples obtained from the subjects. Upon extraction, the DNA was fragmented, and subsequent library preparations were performed. Following sequencing, the obtained DNA sequences were compared against the reference human genome hg19 to assess the coverage and sequencing quality of the targeted regions. The identified variants underwent bioinformatic analysis to evaluate their pathogenicity. The nomenclature used for variant classification adhered to the guidelines provided by the Human Genome Variation Society (HGVS) (http://varnomen.hgvs.org/). Criteria for grading the pathogenic nature of variants were established based on the standards and guidelines for variation interpretation set forth by the American College of Medical Genetics and Genomics (Richards et al., 2015; Kalia et al., 2017). Notably, our methodology may not effectively detect potentially pathogenic variants within gene regulatory regions and deep intronic regions if they consist of deletions or insertions (microvariations) spanning 10 base pairs or less. Moreover, our approach is not optimal for detecting specific types of variations, including dynamic mutations, large segment deletions or duplications, complex recombinations, and genomic structural variations such as inversions, translocations, or rearrangements.

### Model drawing and conservation analysis

The mutated sites within the ZP3 protein were visually depicted using IBS 2.0. Conservation analysis of ZP3 amino acids across various species, including mouse, rat, sheep, bovine, macaque, and human, was conducted using the Align function available on the UniProt website (https://www.uniprot.org/). Schematic representations of both the wild-type (WT) and mutated [ZP3 NM_001110354.2, c.176T>A (p.L59H)] ZP3 proteins were generated using SWISS-MODEL software (https://swissmodel.expasy.org). The 3nk3.1.A.pdb template was selected as the control template for modeling.

### Prediction of mutant protein functions

MutationAssessor (http://mutationassessor.org/) server predicts the functional impact of amino-acid substitutions in proteins, such as mutations discovered in disease. The functional impact is assessed based on evolutionary conservation of the affected amino acid in protein homologs. PolyPhen-2 (http://genetics.bwh.harvard.edu/pph2/ evaluates the impact of mutations on protein function by considering sequence, structure, and conservation information. This tool provides predictions and scores for the impact of mutations on protein function, distinguishing them as harmful, possibly harmful, or benign. By inputting the wild-type ZP3 and ZP3^L59H^ mutant amino acid sequences into these tools and interpreting the results, one can predict the impact of mutations on protein function. A score closer to 1 indicates a higher level of harm.

### Dynamic expression of ZP3

In order to highlight the importance of ZP3 in oocyte and embryo development, we attempted to describe its dynamic expression levels across different species and at various stages of embryo development. The dynamics of *ZP3* mRNA during different embryonic stages (2PN, 2-cell, 4-cell, 8-cell, morula, early ICM, late ICM) across various species were reanalyzed using the single-cell transcriptome database (Boroviak et al., 2018). The dynamic expression of ribosome-bound *Zp3* mRNA in mouse oocytes and embryos was reanalyzed using the mRNA translatomics database (Xiong et al., 2022). The line chart depicting RPF folding represents dynamic changes in ribosome-bound RNA molecules using low-input Ribo-seq methodology (also known as Ribo-lite). By focusing on the gene of interest, one can extract the dynamic expression information (Xiong et al., 2022).

## Results

### Phenotype of patients with ZP3^L59H^ mutations

**FIGURE 1:**
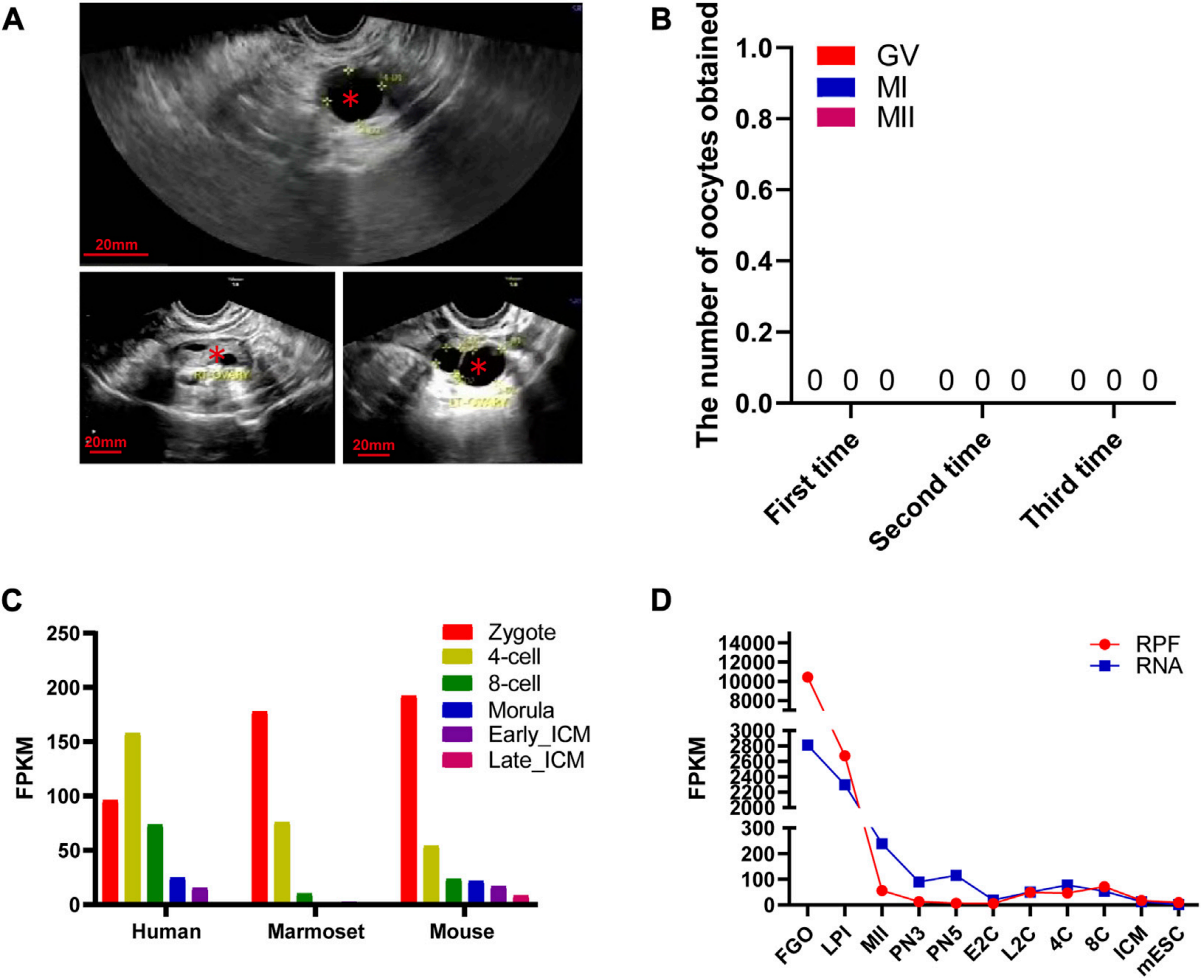
Morphological characteristics of mutant ZP3 follicles and dynamic expression of *ZP3* genes in different species. **(A)** The illustration presents an ultrasound image as a representative sample of a ZP3 mutant follicle. The luminal follicles are denoted by “*”. The scale bar is 20 mm. **(B)** The table shows the number and retrieval period of oocytes obtained from three oocyte retrieval procedures in this particular patient. **(C)** Single-cell RNA sequencing (scRNA-seq) transcriptome analysis exhibits the dynamics of ZP3 mRNA from the zygote stage to the inner cell mass (ICM) stage in mice, marmosets, and humans. **(D)** The dynamic changes in ribosome-bound RNA expression (RPF) of ZP3 mRNA from the oocyte stage to the ICM stage in mice are represented in the graph. The RPF folding line chart illustrates the alterations in RNA molecules bound to ribosomes using low-input Ribo-seq (Ribo-lite). The RNA folding line chart represents regular mRNA sequencing (mRNA-seq). RPF refers to ribosome-protected fragments. Other abbreviations include FGOs (fully grown oocytes), LPI (late prometaphase I), MII (metaphase II), PN3 (early one-cell stage), PN5 (late one-cell stage), E2C (early two-cell stage), L2C (late two-cell stage), 4C (four-cell stage), 8C (eight-cell stage), ICM (inner cell mass of blastocyst), and mESC (mouse embryonic stem cells).

Our center received a 31-year-old female patient presenting with a primary infertility diagnosis of 3 years. No significant abnormalities were observed in her reproductive organs, including the ovaries and uterus, and multiple large follicles were visible (Figure 1A). Interestingly, despite undergoing three oocyte retrieval procedures, no oocytes were obtained (Figure 1B). Hormone levels, including FSH and AMH, were within the normal range for this patient. Conventional semen analysis for her husband showed normal fertility capacity. Due to the patient’s lack of siblings and no other members in the family experiencing fertility disorders within two generations, her case appears to be isolated.

### Expression of human *ZP3*

The human *ZP3* gene is highly expressed in human bipronuclear (2PN) one-cell embryos and shows a further increase in expression at the four-cell stage. Subsequently, the expression level of *ZP3* gradually decreases. These findings suggest that ZP3 plays a crucial role in the development of 2PN embryos. Interestingly, the expression levels of ZP3 in 2PN embryos were most abundant at various stages of preimplantation embryo development in mice and marmosets (Figure 1C). This indicates that the expression of *ZP3* is conserved across different species (Boroviak et al., 2018). Since oocytes store a large amount of reserve mRNA, only mRNA bound to ribosomes can more fully reflect changes in protein expression trends. To further investigate the dynamic changes in ZP3 during oocyte meiosis, ribosome profiling (low-input Ribo-seq) was used to capture *ZP3* mRNA expression, which was found to be 20-fold higher than that in 2PN zygotes (Xiong et al., 2022) (Figure 1D). In comparison to previously reported studies on the regulation of oocyte development by ZP3 (Gao et al., 2017; Jin et al., 2023), our findings contribute to a better understanding of the importance of the ZP3 protein through the following aspects: 1) We have provided a more precise description of *ZP3* mRNA expression levels at the single-cell level. 2) Despite the presence of a large amount of mRNA stored within oocytes, only a fraction of it is translated. Using ribosome profiling (low-input Ribo-seq) methodology, we have further elucidated the dynamic changes in ZP3 protein translation levels. 3) Due to the scarcity of human embryos and ethical constraints, our cross-species analysis has revealed intriguing differences in the expression patterns of the zp3 gene in mice and marmosets. While *ZP3* expression gradually decreases from the embryo to the four-cell and eight-cell stages in these species, *ZP3* gene expression increases in human four-cell embryos. These results suggest species-specific functional differences of the *ZP3* gene, particularly indicating a potentially distinct role for ZP3 in early human embryonic development. Although human embryo samples are limited, this finding is noteworthy and warrants further investigation.

### Impact of ZP3^L59H^ mutations

**FIGURE 2:**
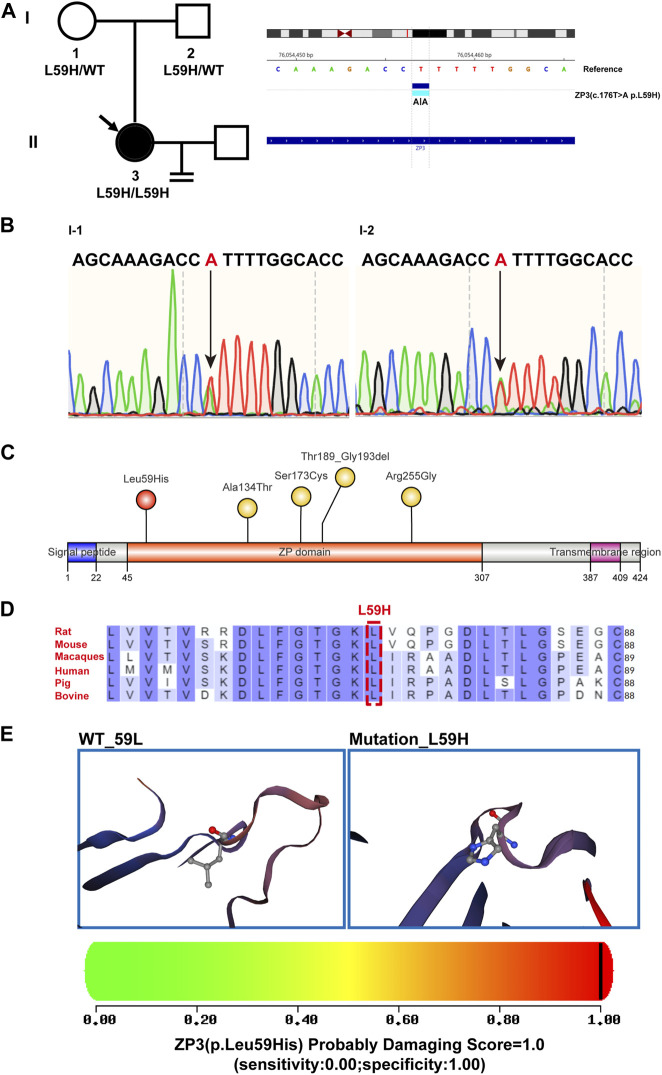
Genealogy and bioinformatics analysis of the proband. **(A)** The pedigree of the patient’s family is depicted on the left, with arrows indicating the proband. The right panel illustrates the mutant ZP3 loci identified through whole-exome sequencing and comparison with the hg19 reference genome. A homozygous variant in ZP3 (NM_001110354.2, c.176T>A (p. L59H)) is highlighted. **(B)** The DNA electropherogram demonstrates the heterozygous state of the 176th nucleotide (T > A) in the ZP3 gene (NM_001110354.2) for both the patient’s father and mother. **(C)** An illustrative diagram showcases the locations of mutated sites within the structural domain of the ZP3 protein. Previously reported sites are indicated in yellow, while the newly identified L59H mutation from this study is highlighted in red. The ZP domain represents the zona pellucida domain. **(D)** The residue L59 within the ZP3 protein is highly conserved across six species. Human-specific sites are marked in red, with a specific emphasis on the L59 position denoted by boxes. **(E)** Utilizing SWISS-MODEL software, spatial structure pattern maps were simulated for both the wild-type and mutant ZP3 proteins. These simulations revealed that the L59H variant alters the protein’s overall shape. PolyPhen-2 was employed to predict the potential impact of the L59H variant on the structure and function of the human ZP3 protein. This mutation was predicted to be potentially damaging, with a score of 1.000 (sensitivity: 0.00; specificity: 1.00).

Through whole exome sequencing, we identified a homozygous mutation in ZP3 (ZP3^L59H^) in this patient with human empty follicle syndrome. Further validation in the patient’s parents revealed that both of them were carriers, with DNA sequencing showing heterozygous ZP3^L59H^ mutations (Figures 2A, B). The ZP3^L59H^ mutations are localized in the zona pellucida (ZP) domain. The ZP domain is a protein polymerization module of approximately 260 amino acids found at the C-terminus of many secreted eukaryotic glycoproteins that play fundamental roles in development, hearing and immunity (Bork and Sander, 1992; Jovine et al., 2002; Yonezawa and Nakano, 2003; Jovine et al., 2004; Al-Awqati, 2008; Bernabeu et al., 2009; Tchatchou et al., 2010). To date, only four mutation sites have been reported that can lead to infertility, all of which are located in the zona pellucida (ZP) domain. These four sites are located within the zona pellucida (ZP) domain of ZP3 (Figure 2C). The ZP3^L59H^ mutation is conserved across six representative species (mouse, rat, sheep, bovine, macaque, and human) (Figure 2D). In comparison to these four known mutation sites, our newly identified mutation occurs at the 59th amino acid residue, which is closest to the N-terminus of ZP domain. Due to this novel mutation, the 59th Leucine (Leu) residue of ZP3 is mutated to Histidine (His). Histidine is a polar amino acid with a positively charged R group, while Leucine is a nonpolar amino acid, exhibiting hydrophobic properties. The mutation at the 59th residue affects the polarity and hydrophobic interactions of the amino acids, resulting in a change in the tertiary structure of the zona pellucida (ZP) domain. Moreover, both PolyPhen-2 and Mutation Assessor predicted high levels of structural damage caused by this mutation, with a score close to 100% (PolyPhen-2: PROBABLY DAMAGING; score 1.000; specificity: 1.00) (Figure 2E).

## Discussion

Oocyte anomalies and female infertility, particularly in the context of Gamete Elastase Factor Syndrome (GEFS), have been associated with various mutations in human ZP genes. In GEFS cases with mutations in ZP1 or ZP3, oocytes were either degenerated or completely absent, while cumulus-oocyte complexes (COCs) could still be obtained (Yang et al., 2021). However, the underlying etiology of GEFS remains unclear. Furthermore, most of the reported mutations have been identified in ZP1, with only a few cases involving ZP3. In this study, we conducted whole exome sequencing on a patient who failed to acquire oocytes despite the presence of follicles after three rounds of ovarian stimulation. We identified a new homozygous variant (NM_001110354.2, c.176T>A (p.L59H)) at the N-terminal end of the ZP domain in the ZP3 gene. Based on further analysis using bioinformatics methods and protein structure modeling, it is suggested that the ZP3^L59H^ mutation may have an impact on the protein’s tertiary structure.

Human ZP glycoproteins (ZPGs) consist of various domains, including an N-terminal signal peptide, a ZP domain (ZPD), a C-terminal peptide with a consensus furin cleavage site (CFCS), and a transmembrane domain (TMD) (Wassarman, 2008). ZPD, especially in ZP3, is a characteristic feature of ZPGs in mice. It has been proposed that ZPDs may independently polymerize into fibrils (Legan et al., 1997; Jovine et al., 2002; Jovine et al., 2006). Mutations in ZP domains, particularly in the N-terminal segment (NTS), have been associated with severe pathologies such as infertility (Al-Awqati, 2008; Wassarman, 2008; Bernabeu et al., 2009; Tchatchou et al., 2010). Intriguingly, the mutation we identified was located in the ZPD of ZP3. Previous studies have linked mutations in ZP3 to impaired assembly of the zona pellucida, resulting in the abnormal assembly of the zona pellucida fiber network and oocyte degeneration associated with GEFS(Chen et al., 2017; Chen et al., 2021; Zhang et al., 2021; Zhang et al., 2022). Additionally, knockdown of ZP3 using specific siRNA has been shown to significantly increase the proportion of oocytes arrested at the GV stage (Gao et al., 2017). Moreover, Zona pellucida 3 (ZP3) has been found to interact with aryl hydrocarbon receptor-interacting protein-like 1 (AIPL1) and lamin A, inhibiting oocyte recovery after meiosis (Jin et al., 2023). These findings suggest that ZP3 may play a role earlier than previously assumed in follicular development or, more directly, in oocyte maturation and meiosis.

Previous studies have mostly focused on the role of ZP3 in oocyte maturation processes (Gao et al., 2017), which is consistent with our analysis showing abnormal expression of ZP3 at different stages of oocyte development (FGO, LPI, MII) (Figure 1D). Considering the abundant expression of ZP3 in oocytes and zygotes (Figures 1C, D), further investigation is warranted to elucidate the specific contributions of ZP3 in the maternal-to-zygotic transition.

The egg envelope is a fibrous matrix composed of conserved components, characterized by shared protein domains. Among mammals, amphibians, birds, and fish, the composition of the egg envelope varies as it is made up of ZP domain-containing proteins (Monne et al., 2006; Goudet et al., 2008; Monne and Jovine, 2011; Meslin et al., 2012). Nevertheless, the nomenclature of ZP glycoprotein subfamilies has historically been complex. As per recent consensus, there are six recognized subfamilies that originated through gene duplication and pseudogenization: ZP1, ZP2/ZPA, ZP3/ZPC, ZP4/ZPB, ZPAX, and ZPD (Goudet et al., 2008). ZP3, which has been evolutionarily conserved across vertebrate lineages, highlights its importance (Goudet et al., 2008). From a protein sequence perspective, the amino acid at position 59 of ZP3 is conserved in humans, mice, and monkeys, among other species, further emphasizing the significance of this site. It is plausible that some ZP genes may have been lost in mammals due to their minimal role in matrix formation and sperm-egg interactions.

In this study, we have elucidated the noteworthy contribution of ZP3^L59R^ mutations to early embryonic development. However, it is important to acknowledge the limitations of our research. Firstly, the sample size employed in this study is relatively small, necessitating the identification of the same mutation site with similar phenotypes across multiple reproductive centers to confirm our findings. Secondly, it is important to consider the inherent limitations of the exome sequencing analysis process, as well as the ongoing updates and improvements to the pathogenic variants database. Consequently, there is a possibility that our study may have missed the discovery of other pathogenic variants in different genes that could have contributed to this phenotype (Richards et al., 2015; Kalia et al., 2017).

In summary, we have reported a novel homozygous variant in ZP3 associated with empty follicle syndrome. This mutation leads to abnormal ZP assembly and follicular development, providing insights into the pathogenicity of the mutation and potential implications for the diagnosis and treatment of EFS.

## Data Availability

The original data presented in the study may be found in the article/Supplementary Material. The data presented in the study are deposited in the National Genomics Data Center (NGDC) repository, accession number PRJCA018168. Further inquiries can be directed to the corresponding authors.
